# Is point-of-care ultrasound disruptive innovation? Formulating why POCUS is different from conventional comprehensive ultrasound

**DOI:** 10.1186/s13089-018-0106-3

**Published:** 2018-10-01

**Authors:** Jesper Weile, Jacob Brix, Anders Broens Moellekaer

**Affiliations:** 1 Emergency Department, Regional Hospital Herning, Herning, Denmark; 2 Research Center for Emergency Medicine, Aarhus University Hospital, Aarhus, Denmark; 3 Department of Learning& Philosophy, Research Group for Evaluation & Capacity Building, Aalborg University, Aalborg, Denmark

**Keywords:** Point-of-care ultrasound, Disruptive innovation, Disruption, Ultrasonography

## Abstract

**Background:**

Point-of-care ultrasound (PoCUS) is spreading throughout Emergency Medicine, Critical Care and Pre-hospital Care. However, there is an underlying inherited conflict with the established specialties performing comprehensive examinations. It has been stated that PoCUS is disruptive innovation. If this is true the definition might open up for a new perspective on differentiating comprehensive ultrasound from PoCUS. PoCUS in the light of disruptive innovation is a different perspective on ultrasound that has not before been academically scrutinized.

**Methods:**

In this paper we investigate if PoCUS is in fact disruptive innovation. This is done by comparative analysis with the point of departure in disruptive innovation theory known from the business world.

**Results:**

We find that a disruptive innovation process is happening. This new knowledge allows us to put forward advice for the stakeholders in the field of ultrasound. It also allows us to challenge the conventional pyramid of expertise used to describe different types of ultrasound. The perspective of this paper is mutual understanding of similarities and differences between conventional and point-of-care ultrasound. Only with this understanding the stakeholders can collaborate and use the full spectrum of ultrasound for the benefit of the patient.

## Background

Point-of-care ultrasound (PoCUS) is different from conventional comprehensive ultrasound examinations—*but what exactly differentiates the two?* Critics would state that PoCUS are merely inferior examinations compared to comprehensive conventional ultrasound examinations performed in an unsuited environment. This standpoint is visualized in the conventional pyramid of expertise, where PoCUS is placed at the bottom [1]. Proponents have stated that PoCUS, “has become a more physiological study than an anatomical one” [2]. These two standpoints represent a disagreement and this conflict does not serve the general purpose of treating every patient to the best of knowledge. Resolving this conflict necessitates a clear theoretical classification between PoCUS and conventional comprehensive ultrasound examinations.

Leaders in ultrasound, such as Resa Lewiss from the University of Colorado School of Medicine, have claimed that PoCUS is disruptive innovation [3]. Clayton Christensen, the father of the disruptive innovation theory, has suggested that healthcare should embrace disruptive innovation [4]. According to Christensen, if a given technology and the business model pertaining to it is undergoing *a process of disruptive innovation*, appreciating this will enable established market players to act and react accordingly [5].

Hence, understanding if PoCUS is undergoing a process of disruptive innovation will aid in a theoretical description of the difference between PoCUS and conventional comprehensive ultrasound examinations and contribute to solving the inherent conflict between conventional comprehensive ultrasound and PoCUS.

To our knowledge it has not been academically investigated if PoCUS adheres to the definition of being true disruptive innovation. This paper will use a comparative analysis, with the point of departure in disruptive innovation theory, to determine whether PoCUS is in fact true disruptive innovation.

If this assumption is true the paper aims first to challenge the conventional pyramid of expertise and second to provide recommendations for stakeholders on how to act or react to the disruptive innovation that is taking place [5].

## Disruptive innovation

**Fig. 1:**
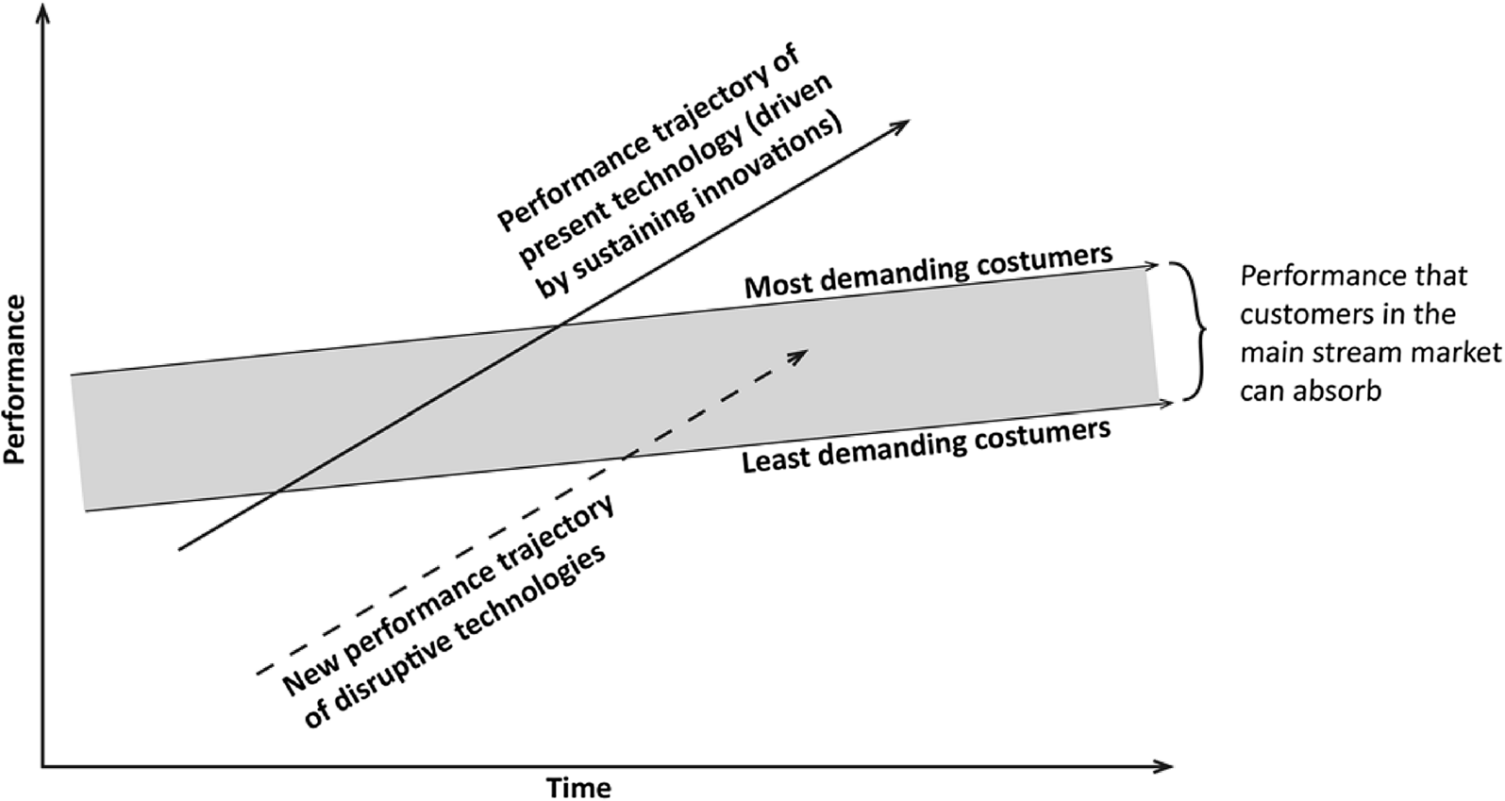
Disruptive innovation. The graph outlines how the disruptive innovation enters the market in it’s own trajectory to the right of the original technology trajectory. Improvement of the original technology within one trajectory is considered sustaining innovation. From Christensen, Clayton M.: The innovator’s dilemma: when new technologies cause great firms to fail (1997)

Disruptive innovation is defined as a process in which a new competitor successfully challenges an established market. The disruptive innovation process can have two different strategies: (1) focus on a low-price alternative to existing technology, where the “job of the product” corresponds with the users’ minimum requirement for relevancy, or (2) focus on creating a new market (potentially in an existing market) in which there is no competition. Hence, disruptive innovation is both different from and in conflict with traditional business models [5, 6]. In 1997, Christensen presented the difference between sustaining and disruptive innovation as shown in Fig. 1 [7]. According to this model the market region’s ability to absorb technology is a developing area ready for increasingly complex technology. Every technology follows a trajectory, which evolves from being below the market, to entering the market, and finally to being developed beyond the customer’s demands. A sustaining innovation process focuses on continuingly delivering products that exceed the current level of customer needs. A disruptive innovation process represents the opportunity for a parallel trajectory, entering the market at the low end of customer demands, where the existing technology’s sustaining innovation has become over-performing and overpriced.

There are many well-known examples of innovative disrupters introducing new platforms with inferior quality at a lower price. One example is how the compact disc (CD) was rapidly pushed from the market after the introduction of the iPod and iTunes Store. In this example, the improvement of CDs, as well as CD players, is the “sustaining innovation.” The compressed files on the iPod were products of inferior sound quality compared to CDs; however, the sound quality was acceptable to meet the minimum requirements of ordinary users listening to music. At the same time, the business model changed from buying a full, physical album to online purchases of either one song or even a full album at a lower price. While the product and the business model changed, despite the inferior quality of the new product, in 2012 there where seven times more purchases of music in iTunes than purchases of physical CDs [8].

## The ultrasound examination as a commodity

The ultrasound examination itself represents value-creation, if regarded as a self-contained commodity. In this case, the patient is a potential customer (e.g., the patient with a sudden onset of dyspnea who aims to purchase an ultrasound examination to investigate cardiac function or an examination of the lungs). The doctors from different specialties are the service providers of the ultrasound examination. Specialties such as radiology and cardiology can be considered the “established service providers” of comprehensive conventional ultrasound examinations, and the “new suppliers” of ultrasound examinations would be specialties in which the simple PoCUS examination has become relevant such as anesthesiology or emergency medicine. In PoCUS multiple abbreviated protocols have been proposed. The general idea of these is abbreviation of a corresponding comprehensive examination. Examples of this are protocols such as FEEL (Focused Echocardiographic Evaluation in Life Support) [9], FEER (Focused Echocardiographic Evaluation in Resuscitation [10] or FATE (Focus Assessed Transthoracis Echocardiography) [11] vs. the minimum requirements for comprehensive echocardiography protocol put forward by leading medical societies [12, 13]. The abbreviated examination aims to be simpler, but faster and with a much wider indication.

Hence, the PoCUS examination could represent the creation of a new market in an existing market. This is argued because PoCUS can be performed anywhere with any type of ultrasound machine, and consequently, the hardware technology itself is less important. This is a new business model and an opposite approach to ultrasound examination compared to comprehensive conventional echocardiography or a comprehensive conventional radiological ultrasound examination; PoCUS brings the ultrasound to the patient, as opposed to the patient being referred and transported to the ultrasound examination room hence making the examination available at all times.

In summary, the PoCUS examination meets the criteria of an occurring process of disruptive innovation:

1. The examination is inferior compared to what exists on the market (e.g. eyeballing four cardiac windows in PoCUS protocols vs. extensive views and qualitative measurements on flow and left ventricular ejection fraction in the comprehensive echocardiography protocol).
2. The examination is at a lower cost (time equals cost) compared to existing technology.
3. The PoCUS examination follows the dogma of a different business model (the care giver provides the product instantly at the bedside).

## Challenging the pyramid of expertise

**Fig. 2:**
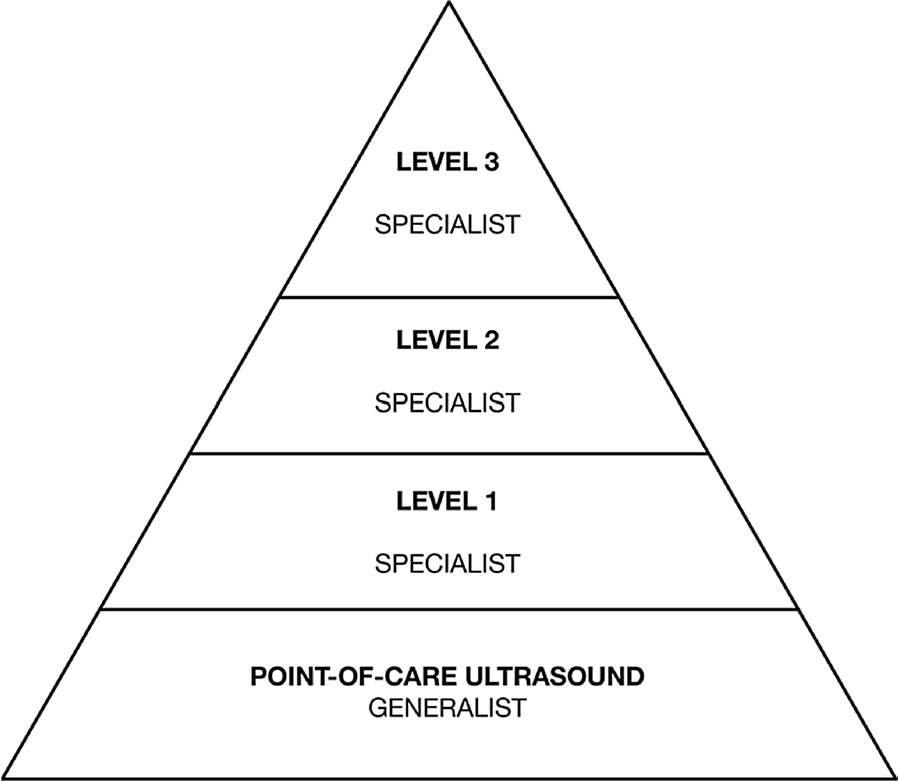
The expertise pyramid. The conventional perception of ultrasound competency expressed in the expertise pyramid. The point-of-care examination is placed as the lowest level of expertise, and the pyramid visualizes how the physician can extend their expertise to advance to more sophisticated levels. The underlying assumption is that PoCUS is an inferior examination. The pyramid does not permit point-of-care ultrasound to possess strengths, such as speed, cost, and availability, which are superior to the layers above it (Adapted from Oxorn and Pearlman [1])

In the conventional perception of ultrasound, the PoCUS examination is placed at the bottom of an expertise pyramid as shown in Fig. 2. In this view, the pyramid is a learning ladder that one climbs from the bottom to the top by acquiring expertise [1].

PoCUS is based on the premise that the examinations are acceptable for their intended purpose but inferior in quality when compared to comprehensive examinations. However, the PoCUS examinations excel in speed and availability for every patient and are as accessible as the stethoscope. The conventional pyramid is static and leaves no room for PoCUS to offer superiority on any parameter compared to comprehensive examinations. If the physician performing PoCUS rises to a higher level of expertise in the pyramid, speed is lost, as the examiner extends the exam toward something more comprehensive and time consuming. Availability is also lost, as only a few doctors (who are not always present in the department) can be expected to perform the examination at the expert level. Hence climbing the pyramid of expertise also weakens the product.

**Fig. 3:**
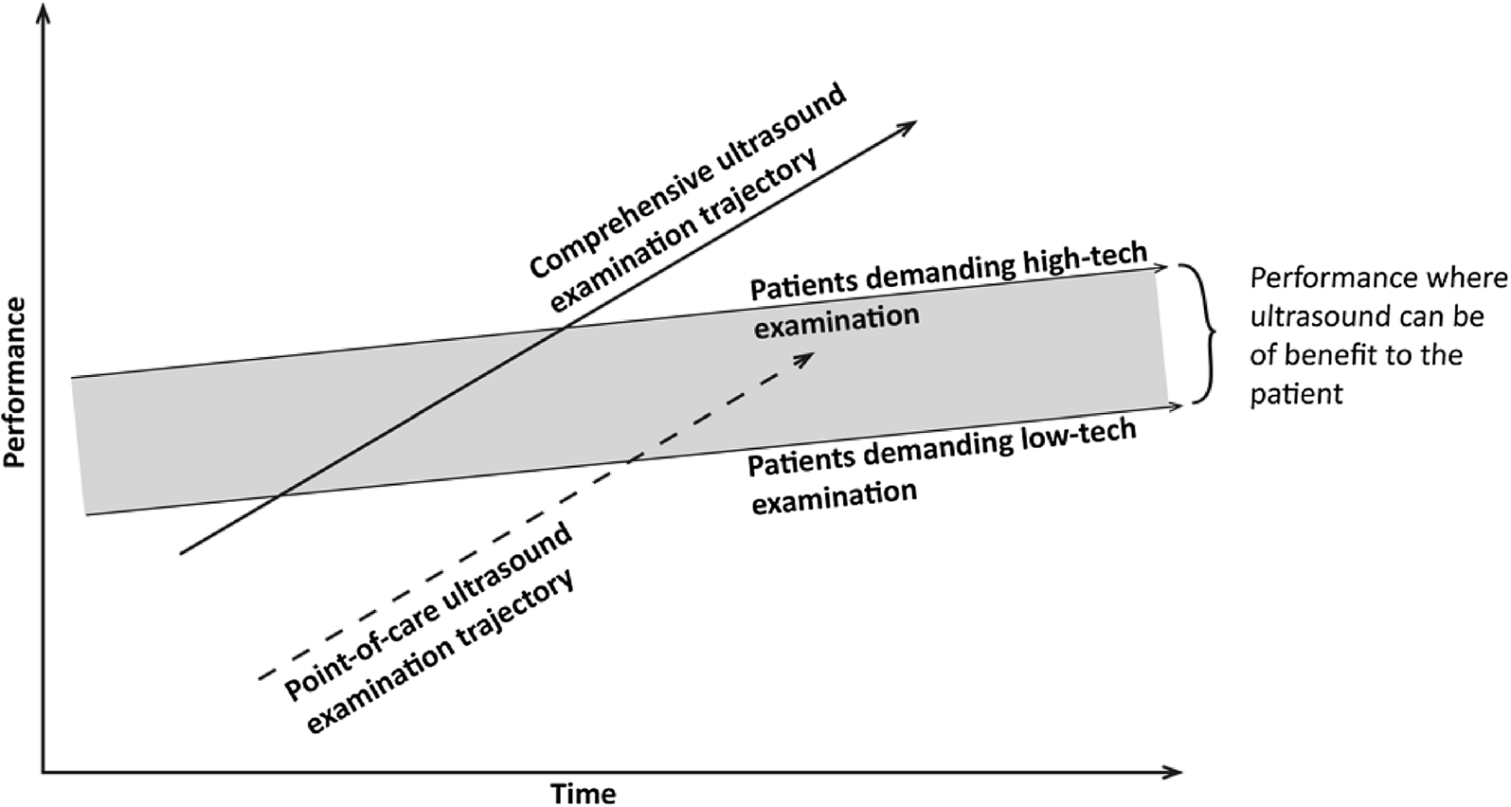
Point-of-care Ultrasound Expressed as a Disruptive Innovation. In this context, the conventional comprehensive ultrasound examination and the point-of-care examination are two parallel trajectories. PoCUS examination is a disruptive innovation, as its speed, cost, and availability differ from that of the conventional comprehensive examination. The speed and availability enable the examination to become an extension to any physical examination at any time. The point-of-care examination is inferior to comprehensive examinations, but meets the low end of customer demand (Adapted from Christensen [7])

Applying the theory of disruptive innovation to the PoCUS examination can be presented as shown in Fig. 3. Here, the comprehensive ultrasound examination and the PoCUS examination are perceived as two parallel trajectories. The trajectory of the comprehensive examination will reflect the level of expertise: higher expertise is considered higher performance. This places the level-3 expert sonographer from the pyramid in Fig. 2 at the top of (or maybe above) the market region’s ability to absorb technology. In this presentation, levels 2 and 1 will follow further down on the same trajectory. PoCUS is placed on its own trajectory, as the examination is fast and available but inferior in range. Depending on the examiner’s skill, the examination performed will be placed somewhere along the trajectory. Due to high availability, the indication for the examination becomes wider, resembling a much larger customer base; however, the high-end customer will still exist and demand highly specialized examinations.

## How stakeholders should act and react

For all medical specialties performing ultrasound or PoCUS, it is paramount to understand the differences between the services.

For the disrupter, this means understanding the advantages but also the limitations of PoCUS. Hence, the disrupter should be cautious in evolving PoCUS toward the standards of comprehensive examinations, as the examination will lose speed and availability. The time-consuming comprehensive examinations will obstruct patient flow.

The disrupted (e.g., cardiology or radiology) must focus on the core values of the high-quality product and cultivate these. The disrupted parties should also promote knowledge of the potential gains from a conventional examination over a PoCUS examination. This will enable providers of PoCUS to identify the highly demanding customer and recognize when referrals can create value for the patient. At the same time, the disrupted parties should find delight in the fact that even more patients will now have the privilege of ultrasound examinations performed.

Aspiring to increase quality should also inspire to increase standards of education for providers. The PoCUS examinations are faster and simpler; however, this does not necessarily mean they are easier to master. The providers must receive appropriate training to avoid overconfidence and potential wrongful interpretation. The commodity must stay above the low end of customer demands (Fig. 3) or it loses its definition as a disruptive innovation.

## Conclusion

PoCUS examination is in a process of disruptive innovation. This knowledge provides a theoretical basis to segregate PoCUS from comprehensive ultrasound examinations.

Perceiving PoCUS as a disruptive innovation challenges the conventional view of the PoCUS examination being inferior on the expertise pyramid. This perspective leads to viewing PoCUS and conventional ultrasound as two parallel trajectories with different strengths, opportunities, and patients.

The innovation is vulnerable to up-market movements due to the physician performing over the means of existence, with the disruptive innovation losing the strengths of speed and availability. The innovation is also vulnerable to down-market movement if the performing physician does not possess sufficient competency.

The disrupter and the disrupted should both embrace the similarities and differences in the comprehensive specialized examinations and PoCUS. The core values of each examination should be recognized and cultivated [5, 6].
